# Circulating microRNAs as biomarkers of Chagas cardiomyopathy

**DOI:** 10.3389/fcvm.2023.1250029

**Published:** 2023-12-19

**Authors:** Laura Antonietti, Javier Mariani, María Jose Martínez, Manuela Santalla, Natalia Vensentini, Diego Alfredo Kyle, Maximiliano de Abreu, Carlos Tajer, Ezequiel Lacunza, Paola Ferrero

**Affiliations:** ^1^ Department of Cardiology, El Cruce Hospital, Florencio Varela, Buenos Aires, Argentina; ^2^ Health Sciences Institute, Arturo Jauretche National University, Florencio Varela, Buenos Aires, Argentina; ^3^ Cardiovascular Research Center Dr. Horacio Cingolani, Faculty of Medical Sciences, La Plata National University, La Plata, Buenos Aires, Argentina; ^4^ Basic and Applied Inmunological Research, Faculty of Medical Sciences, La Plata National University, La Plata, Buenos Aires, Argentina

**Keywords:** Chagas disease, Chagas cardiomyopathy, microRNAs, circulating microRNAs, biomarkers

## Abstract

**Background:**

Chagas cardiomyopathy (CHCM) is the most important clinical manifestation of Chagas disease. The analysis of cardiac miRNAs may contribute to predicting the progression to CHCM in Chagas indeterminate phase and/or to the differential diagnosis for cardiomyopathy.

**Methods:**

We carried out a case-control study to identify circulating miRNAs associated with CHCM. We assigned 104 participants to four groups: healthy controls (HC), Chagas non-cardiomyopathy controls, CHCM cases, and ischemic cardiomyopathy controls. We performed a clinical, echocardiographic, and laboratory evaluation and profiled circulating miRNA in the serum samples.

**Results:**

Differences between groups were observed in clinical variables and in the analysis of miRNAs. Compared to HC, CHCM participants had 4 over-expressed and 6 under-expressed miRNAs; miR-95-3p and miR-130b-3p were upregulated in CHCM compared with controls, Chagas non-cardiomyopathy and ischemic cardiomyopathy participants, suggesting that might be a hallmark of CHCM. Analysis of gene targets associated with cardiac injury yielded results of genes involved in arrhythmia generation, cardiomegaly, and hypertrophy.

**Conclusions:**

Our data suggest that the expression of circulating miRNAs identified by deep sequencing in CHCM could be associated with different cardiac phenotypes in CHCM subjects, compared with Chagas non-CHCM, ischemic cardiomyopathy controls, and healthy controls.

## Introduction

Chagas disease is an endemic parasitosis in Latin America, spreading to other regions due to the migration of infected populations. Currently, six to eight million people have *Trypanosoma cruzi* infection, and an additional 70 million are at risk of infection (1).

The clinical course of chronic Chagas disease is variable. In most cases, it is asymptomatic, with no changes in the EKG nor structural or functional myocardial disturbances—considered an “indeterminate” form (2, 3). One-third evolve with symptoms of CHCM (4), characterized by a continuum of cardiac damage that can lead to global ventricular dysfunction and progressive heart failure (3), and it is associated with premature mortality (4–6).

A relevant diagnostic challenge is to identify which subjects with Chagas disease will progress to CHCM, to promote an appropriate monitoring and timely therapeutic interventions. Several biomarkers of diagnostic and/or prognostic value of the disease have been identified (7–13), but they are useful for risk stratification when CHCM is already established.

In recent years, the study of circulating plasma miRNAs has highlighted their role as potential markers for predicting the risk of CHCM progression (14). miRNAs are small ribonucleic acid molecules associated with the regulation of gene expression (15). They are involved in all cellular processes, and their expression profiles differ depending on physio-pathological conditions (16). miRNAs are expressed throughout development in various tissues, including the cardiovascular system (17). They are involved in intercellular communication and between different organs, so they can be found circulating in the blood (18, 19).

The miRNAs are essential in the regulation of cardiovascular biology (17, 20, 21). Circulating miRNAs have been studied as potential biomarkers for the diagnosis and prognosis of some cardiovascular diseases due to their stability and easy detection (22, 23). Several studies have detected changes in their expression in heart failure, myocardial infarction, atherosclerotic disease and arrythmia (23–26).

Our hypothesis sustains that evaluating circulating cardiac miRNAs as biomarkers may constitute a precision medicine tool to predict the potential evolution towards CHCM in patients with Chagas disease in its indeterminate phase and/or contribute to the differential diagnosis of cardiomyopathy in Chagas subjects.

The aim of this study was to identify circulating miRNAs associated with CHCM.

## Material and methods

We carried out an observational, case-control, single-center study.

### Study population

**Figure 1:**
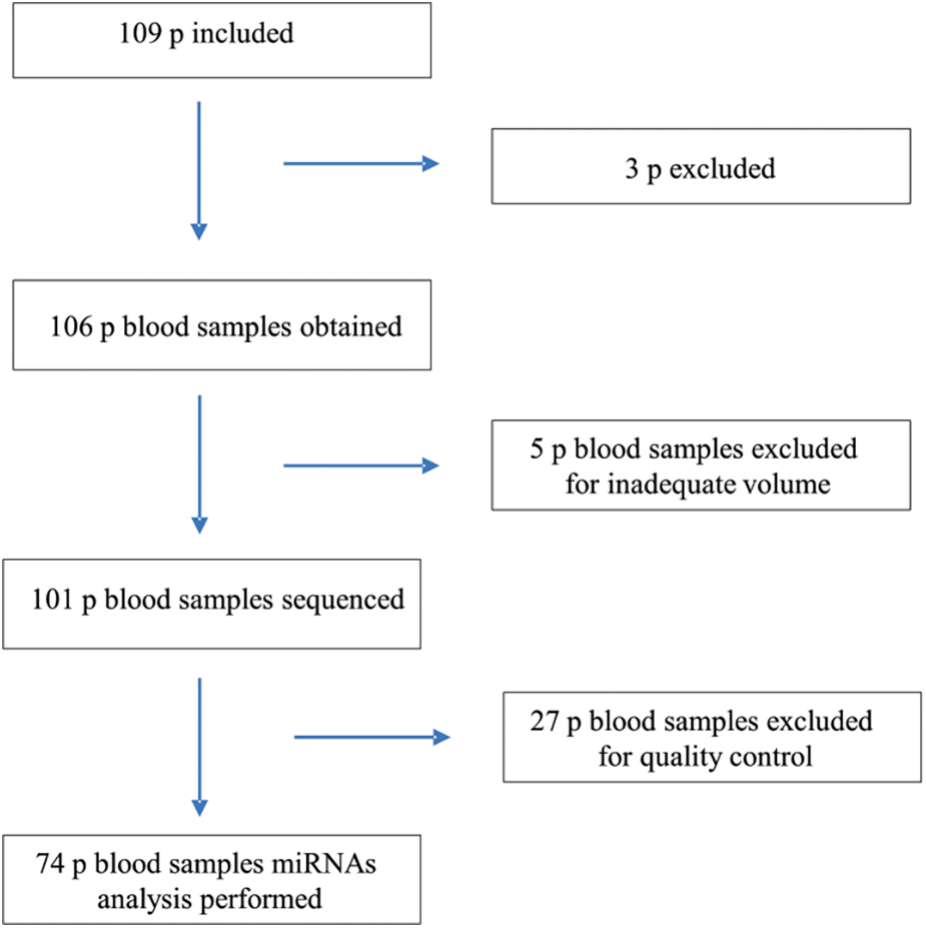
Flowchart of the inclusion process and study methods.

After giving their consent, 109 participants aged between 18 and 85 years were enrolled and underwent a clinical evaluation, a blood sample test, an electrocardiogram (EKG) and a transthoracic echocardiogram (TTE) in all cases (Figure 1).

Serology for Chagas disease was confirmed by two methods: automated chemiluminescence assay (ELISA) and rapid immunochromatographic test. In inconclusive cases, a gelatin particle agglutination test was performed (27). The absence of cardiomyopathy was defined by no rhythm disturbances or conduction system abnormalities on EKG, left ventricular ejection fraction (LVEF) ≥55%, and no other pathologic findings.

After the baseline visit, participants were assigned to one of four groups (G), according to the inclusion criteria:

- G1 (HC): Twenty (20) healthy controls had negative Chagas test, asymptomatic, and absence of heart disease.
- G2 (Chagas non-CHCM): Twenty-four (24) Chagas non-cardiomyopathy controls characterized by positive Chagas test and absence of cardiomyopathy.
- G3 (CHCM): Fourteen (14) Chagas cardiomyopathy cases were defined by positive Chagas test, LVEF less than 45%, and history of symptoms or hospitalization for heart failure. The determination that the cardiomyopathy is secondary to Chagas disease was established according to medical criteria, considering the most prevalent signs of the disease, based on the serology results and the clinical evaluation: absence of coronary artery disease (CAD) history, common Chagas EKG and/or echocardiographic signs. The EKG signs considered were: complete right bundle branch block (RBBB), left anterior fascicular block (LAFB), a combination of complete RBBB/LAFB, AV node conduction abnormalities or arrhythmias. The echocardiographic signs considered were left ventricular apical, non-segmentary wall abnormalities, and left ventricular ejection fraction <45%. Other EKG/echocardiographic signs were not considered in order to improve the specificity in the inclusion of CHCM cases.
- G4 (iCM): Sixteen (16) ischemic cardiomyopathy controls, defined by negative Chagas test, CAD documented by non-invasive multi-slice computed tomography (CT) coronary angiography, LVEF <45%, and history of symptoms or hospitalization for heart failure.

Pregnant women, subjects with any cardiomyopathy (hypertrophic, hypertensive, infiltrative, valvular), or chronic renal failure on dialysis were excluded. Patients whose echocardiographic findings could explain heart failure from another cause, such as valvular disease, were excluded. Participants had to be stable at the moment of inclusion, at least 30 days after hospitalization, for any cause. Angiography or non-invasive multi-slice TC was not routinely performed in all participants, except those with a history of CAD, who were assigned to the iCM group once the diagnosis was confirmed.

#### RNA isolation

miRNA extraction was performed using the miRNeasy Serum/Plasma Advanced Kit (Qiagen GmbH, Hilden). Samples for miRNA analysis were stored at −80°C until further processing. Subsequently, sequencing was carried out on an Illumina HiSeq 2000 instrument at the University of Heidelberg, Germany. Out of the total samples collected, 101 samples underwent sequencing. Prior to sequencing, five blood samples were excluded due to inadequate volume. Following quality control procedures, 27 samples were excluded from the analysis. The resulting expression matrix consisted of 2,656 miRNAs and a total of 74 samples. These samples were categorized into four study groups: G1 represented healthy controls (HC) (*n* = 20); G2 comprised Chagas-positive individuals without heart disease (Chagas non-CHCM) (*n* = 24); G3 consisted of Chagas-positive individuals with cardiomyopathy (CHCM) (*n* = 14); and G4 consisted of Chagas-negative individuals with ischemic cardiomyopathy (iCM) (*n* = 16).

#### Bioinformatic analysis

To identify differentially expressed miRNAs between the different comparisons: G1 (HC) vs. G2 (Chagas non-CHCM); G1 (HC) vs. G3 (CHCM); G1 (HC) vs. G4 (iCM); G2 (Chagas non-CHCM) vs. G3 (CHCM), and G3 (CHCM) vs. G4 (iCM); we utilized the DESeq2 package in R/Bioconductor (28). A LogFC >|0.6| and an adjusted *p*-value <0.05 were established as significance criteria. A list of experimentally validated target genes derived from the differentially expressed miRNAs from the group comparison G1(HC) vs. G3 (CHCM) was obtained. We used DIANA tools—Tarbase v8 (uth.gr) for that purpose. Only those targets identified by high-throughput methodologies and with a score value >0.5 according to the filtering criteria provided by the web resource were considered. To investigate which target genes have been related to heart disease, we downloaded gene signatures associated with cardiomyopathies from GSEA (http://www.gsea-msigdb.org/) (29, 30).

#### Statistical analysis

According to their distribution, continuous variables were expressed as mean and standard deviation or median and interquartile range (IR). Categorical variables were expressed as numbers and percentages. Differences between groups for continuous variables were evaluated with T-test or ANOVA for comparison between two or more groups, respectively, or the Kruskal–Wallis test, according to their distribution. Categorical variables were compared using the Chi2 test and Fisher's test. For the identification of differentially expressed (DE) miRNAs between groups, the Deseq2 algorithm was used in the R statistical environment. Focusing on miRNAs with a log-fold change (LogFC) greater than |0.6| and an adjusted *p*-value (*p* adj) less than 0.05 to identify significant differentially expressed miRNAs. This threshold was chosen to ensure a stringent criterion for statistical significance. The *p*-values for the association between the miRNA's regulation and the group of the participants were adjusted using the Bonferroni method. We used Sankey plots to construct the networks representing the miRNA-Target-Cardiomyopathy gene signature. Correlation analysis was performed using appropriate statistical methods to assess the relationships between miRNA expression and clinical variables. The “corrplot” package in R was employed to generate correlation plots, visualizing the strength and direction of correlations. Quantitative Venn diagrams were made with the FunRich tool (http://funrich.org/) (31). ROC curves were constructed for upregulated miRNAs, and the area under the ROC curve was calculated from the relative expression values of the miRNAs in the Chagas cardiomyopathy group. All analyses were conducted using R version 4.2.2 (R: A language and environment for statistical computing. R Foundation for Statistical Computing, Vienna, Austria).

## Results

Between November 2016 and March 2020, 109 subjects were enrolled in this study. Three patients were excluded: two were screening failures, and one participant did not complete the baseline visit; 74 patients were included in the sequencing analysis (Figure 1).

### Clinical description of the population

Table 1 shows the demographic and clinical characteristics of the participants at the baseline. Comparing cardiomyopathy groups, sinus rhythm tended to be more common among iCM participants, and a prior diagnosis of arrhythmias was higher in CHCM participants. In non-cardiomyopathy groups, Chagas controls presented a significantly wide QRS complex. Participants with CHCM and iCM had increased left ventricular diastolic diameter (LVDD), left ventricular mass index (LVMI), and depressed LVEF. Although 3 cases with atrial fibrillation or flutter and 8 cases with ventricular arrhythmia history were identified in participants with CHCM, none were of such severity that could lead to a reduction in ejection fraction, and all participants were stable at the time of the study entry.

**Table 1:** Clinical description of the population.

Variable	G 1 (HC) *N* = 20	G 2 (Chagas) non-CMCH) *N* = 24	G 3 (CHCM) *N* = 14	G 4 (iCM) *N* = 16	*p*
Age, media (SD)	42.7(+/−10.9)	53.6 (+/−10.6)	56 (+/−8.6)	57.0 (+/−7.2)	<0.01*
Female % (*n*)	65 (13)	66.7 (16)	14.3 (2)	12.5 (2)	<0.01**
Medical history					
Hypertension % (*n*)	30 (6)	41.7 (10)	14.3 (2)	50 (8)	0.2**
Dislipemia, % (*n*)	20 (4)	33.3 (8)	21.4 (3)	62.5 (10)	0.04**
Diabetes, % (*n*)	5.3 (1)	12.5 (3)	7.1 (1)	25 (4)	0.3**
Smoking present or former, % (*n*)	60 (12)	48.8 (11)	35.7 (5)	81.2 (13)	0.03**
History of any arrythmia % (*n*)	5 (1)	4.2 (1)	78.6 (11)	31.2 (5)	<0.01**
History of ventricular arrythmia % (*n*)	0	0	42.9 (6)	18.8 (3)	<0.01**
EKG data					
Sinus rhythm % (*n*)	100 (20)	95.8 (23)	50 (7)	93.8 (15)	<0.01**
HR, median (IR)	68 (58–75)	65 (60–75)	59 (52.5–68.7)	70 (62–73)	0.2**
QRS, median (IR)	80 (80–80)	80 (80–100)	160 (100–160)	120 (95–145)	<0.01**
Echocardiographic data					
LVEF, % (IR)	65 (63–69)	63 (59–68.5)	31 (24–37.5)	27.5 (24.5–32.2)	<0.01***
LVSD, mm (IR)	44 (41.1–48.8)	46.3 (43.9–48.7)	654.2 (58.6–68.5)	67.7 (60.2–72.4)	<0.01***
LVMI, g/m^2^ (IR)	62.5 (56.5–73.2)	72.5 (67.7–80)	112 (99–160)	118 (116–136)	<0.01***

HC, healthy controls; CHMC, Chagas cardiomyopathy; iCM, ischemic cardiomyopathy; SD, standard deviation; HR, heart rate; IR, interquartile range; LVEF, left ventricular ejection fraction; LVDD, left ventricular diastolic diameter; LVMI, left ventricular mass index.

* *p*-value for ANOVA test.

** *p*-value for Fisher's test.

*** *p*-value for Kruskal–Wallis test.

### Differentially expressed miRNAs

**Figure 2:**
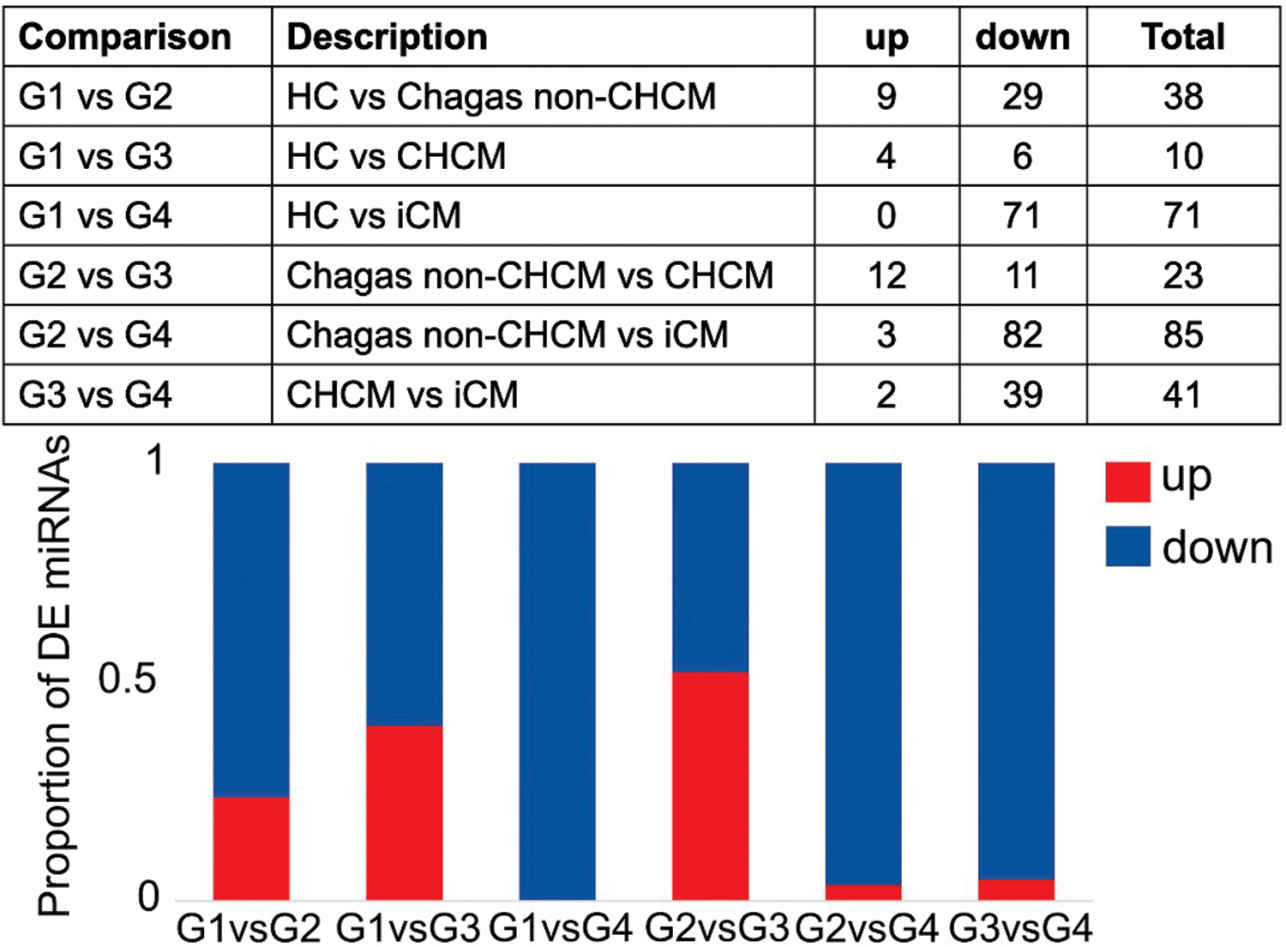
Differential expression analysis of microRNAs (miRNAs). The figure provides a comprehensive overview of the results obtained from the differential expression analysis of microRNAs (miRNAs). The upper panel shows the distribution of differentially expressed (DE) miRNAs across the comparisons between groups: G1(HC); G2 (Chagas non-CHCM) (LogFC >|0.6|; *p* value < 0.05); G3 (CHCM); G4 (iCM). The lower panel features a stacked bar plot, offering a visual representation of the proportion of upregulated and downregulated miRNAs within each comparison. Each stacked bar is segmented to depict the relative contributions of upregulated and downregulated miRNAs.

We characterized circulating miRNAs in the serum of all groups of patients using a deep-sequencing approach. Compared to G1 (HC), we observed differential expression of 38 miRNAs in G2 (Chagas non-CHCM), 10 miRNAs in G3 (CHCM), and 71 miRNAs in G4 (iCM). The profile of miRNAs of G3 (CHCM) subjects showed 76% of downregulated and 24% overexpressed miRNAs. In contrast, in G4 (iCM) we found 100% downregulated miRNAs. G3 (CHCM) participants showed 23 differentially expressed miRNAs compared to those of G2 (Chagas non-CHCM), while G4 (iCM) participants showed 85 miRNAs dysregulated. Both cardiomyopathies—G4 (iCM) vs. G3 (CHCM)- exhibited 41 miRNAs differentially expressed (Figure 2, Supplementary File S1).

**Figure 3:**
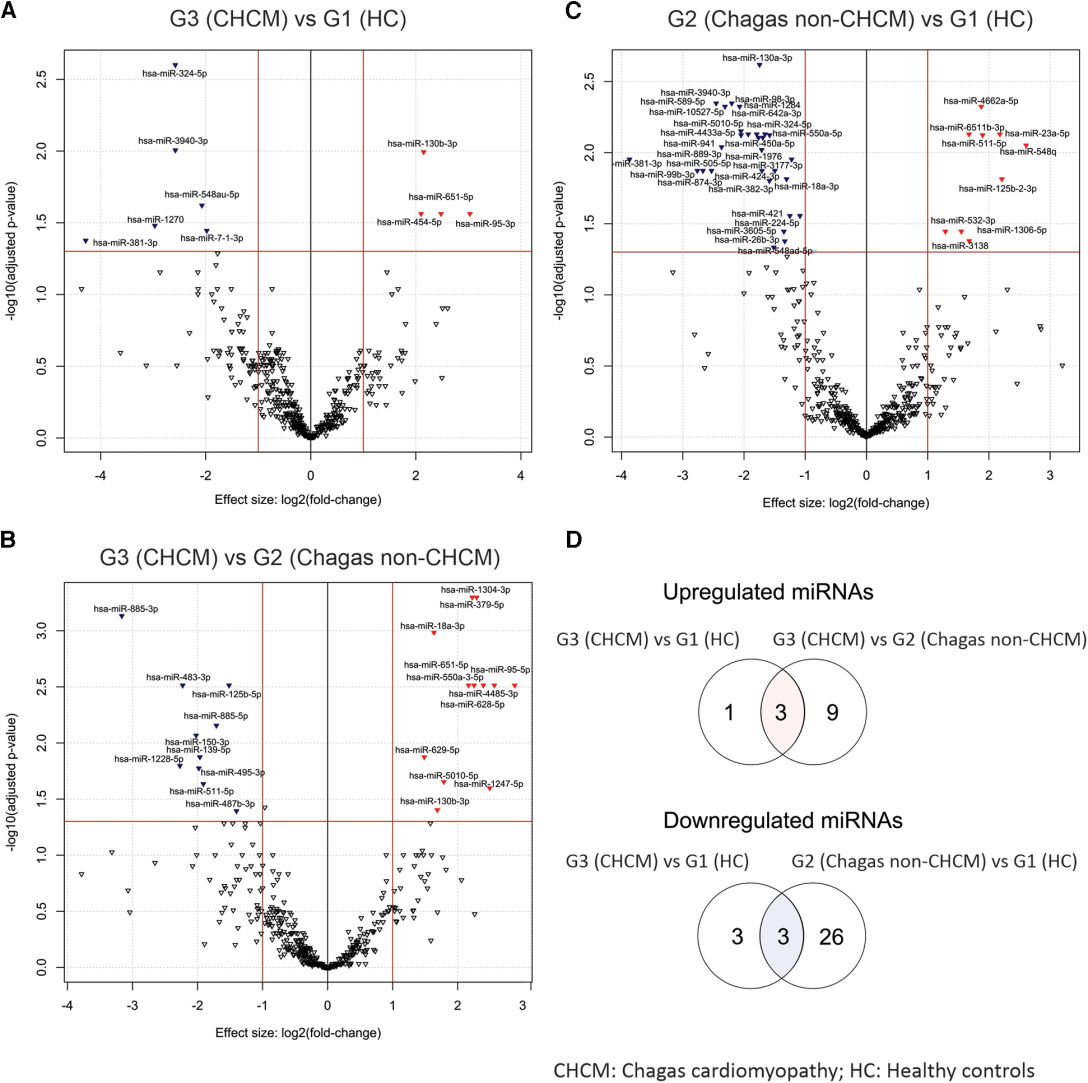
Volcano plots illustrating differentially expressed circulating miRNAs. The volcano plots depict the distinct profiles of differentially expressed circulating miRNAs derived from the following comparisons: (**A**) G3 (CHCM) vs. G1 HC, (**B**) G3 (CHCM) vs. G2 (Chagas non-CHCM), and (**C**) G2 (Chagas non-CHCM) vs. G1 (HC). In each plot, the y-axis represents the negative base-10 logarithm of the *p*-values, while the x-axis reflects the fold change, presented as a log2-transformed ratio of expression between the groups being compared. Distinctive red and blue arrowheads denote significantly upregulated and downregulated miRNAs, respectively, within the initial group of each comparison (with LogFC >0.6 and *p*-value < 0.05). (**D**) Venn diagrams showing the shared upregulated miRNAs between G3 (CHCM) vs. G1 (HC) and G3 (CHCM) vs. G2 (Chagas non-CHCM) comparisons (upper diagram, **D**) and the shared downregulated miRNA between G3 (CHCM) vs. G1 (HC) and G2 (Chagas non-CHCM) vs. G1 (HC) comparisons (lower diagram, **D**).

Figure 3 shows details of the compared profile of the miRNAs obtained from the serum of participants. Figure 3A is a volcano plot of miRNAs from G1 (HC) and G3 (CHCM), while Figure 3B compares G2 (Chagas non-CHCM) and G3 (CHCM) participants. Figure 3C compares G1 (HC) and G2 (Chagas non-CHCM) participants. Three upregulated miRNAs (miR-130b-3p, miR-651-5p, and miR-95-3p) were overexpressed in G3 (CHCM) as compared to G1 (HC) and G2 (Chagas non-CHCM) (Figure 3D). G3 (CHCM) participants shared three downregulated miRNAs with G2 (Chagas non-CHCM) (miR-381-3p, miR-324-5p, and miR-3940-3p) (Figure 3D). Both miR-381-3p and miR-324-5p were downregulated in G3 (CHCM) as well as in G4 (iCM). However, miR-3940-3p was downregulated only in G3 (CHCM) participants. Furthermore, miR-95-3p and miR-130b-3p were downregulated in G4 (iCM) subjects as compared with G3 (CHCM) (Supplementary File S1). Therefore, miR-95-3p and miR-130b-3p were upregulated in CHCM compared to all other groups.

### Predicted target genes of the Chagas cardiomyopathy associated miRNAs

**Figure 4:**
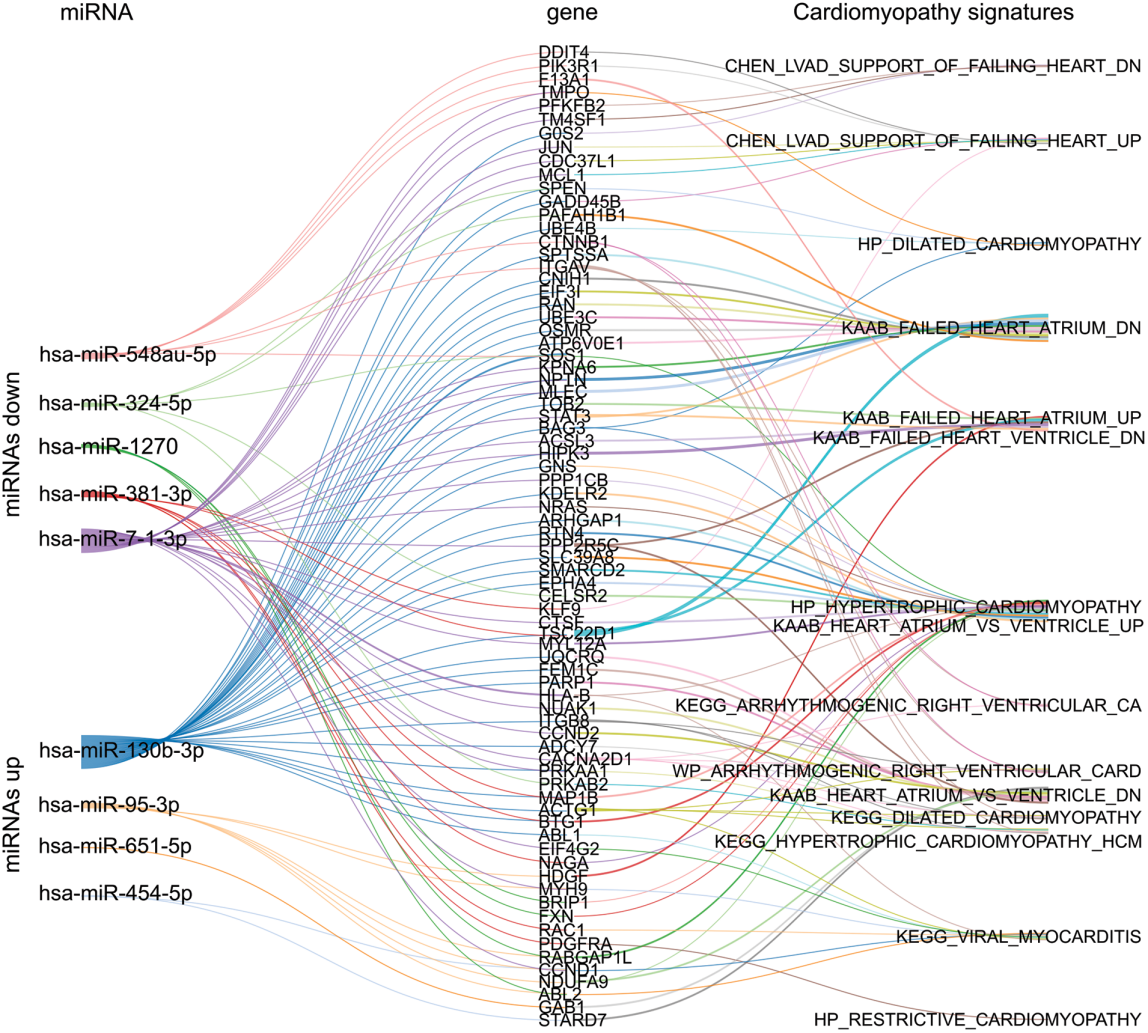
Sankey plot network between selected miRNAs, gene targets, and cardiomyopathy signatures. The network illustrates the relationships between the up and downregulated miRNAs of G3 (CHCM), their gene targets, and the subsequent connections among these genes in the context of cardiomyopathies signatures sourced from MolSigDB.

We aimed to identify potential genes that may be associated with cardiomyopathies in G3 (CHCM) participants. We first predicted the target messenger RNAs (mRNAs) for both upregulated and downregulated miRNAs identified in G3 (CHCM) and compiled a list of these genes. We then searched for cardiomyopathy signatures within the MolSiDB database to identify genes that are known to be associated with hypertrophy, dilated cardiomyopathy, heart failure, arrhythmias, and myocarditis (Supplementary File S2). We found several of miRNAs target genes that fit these criteria and may be important for further investigation (Figure 4).

**Figure 5:**
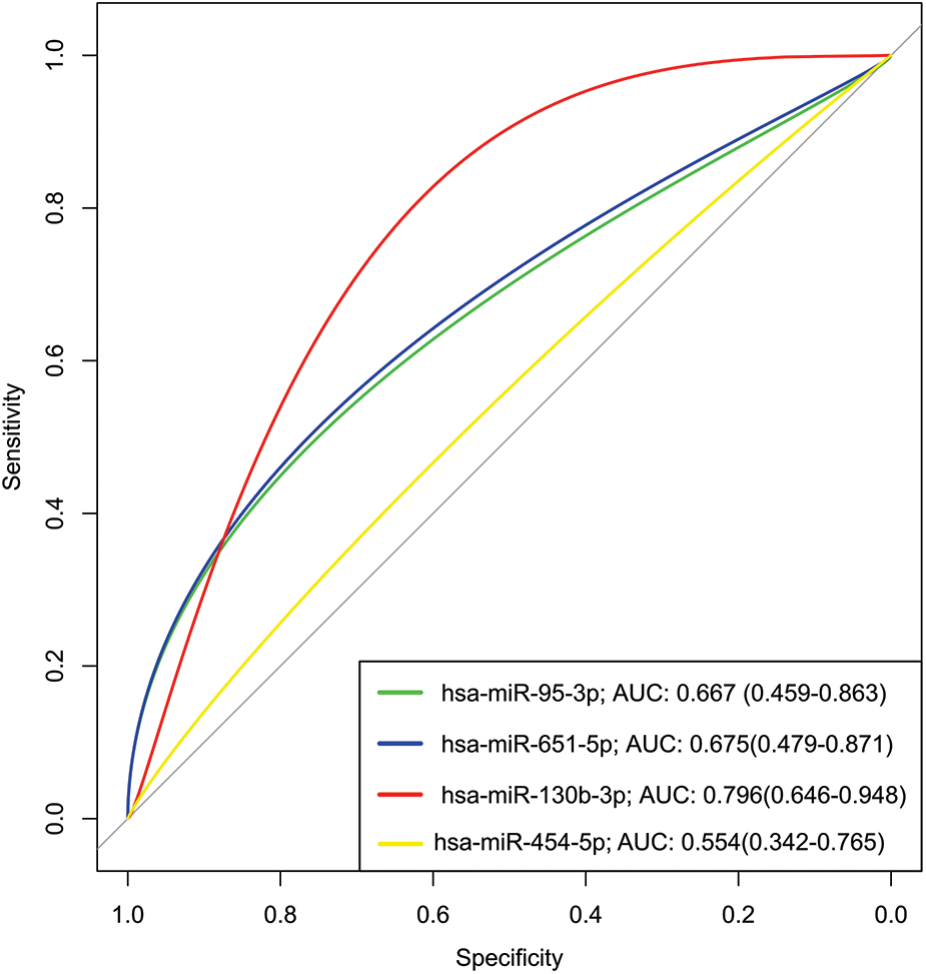
Receiver operating characteristic (ROC) analysis of upregulated miRNAs in serum from chronic chagas disease patients. A Receiver Operating Characteristic (ROC) analysis was conducted to evaluate the performance of upregulated miRNAs as potential biomarkers for chronic Chagas disease. The Area Under the Curve (AUC) was calculated for each miRNA to assess its discriminatory power. Higher AUC values indicate stronger discriminatory ability, suggesting that miR-130b-3p (AUC >0.7) shows promising potential as a diagnostic biomarker for chronic Chagas disease. The ROC analysis underscores the utility of these miRNAs in distinguishing disease status, contributing to improved clinical management.

To assess the predictive efficacy of the upregulated miRNAs within the G3 (CHCM) group, we used receiver operating characteristic (ROC) curves. Our analysis showed the remarkable performance of these miRNAs as predictive tools [area under the curve (AUC) value spanning from 0.55 for miR-454-5p to 0.79 for miR-130b-3p] (Figure 5). The miR-130 had a superior discriminatory value, marked by the higher AUC value. These findings support the potential of miR-130 as a promising biomarker for diagnosis and prognostic of cardiomyopathy in Chagas disease. Although these outcomes are promising, further validation studies are needed to confirm the potential clinical applications of miR-130.

To further explore the potential relevance of miR-130 and miR-95 target genes in the pathogenesis of cardiomyopathy, we conducted an analysis focused on identifying those genes specifically associated with atrial and/or ventricular arrhythmias. Our rationale for this analysis was based on the well-established link between arrhythmias and cardiac dysfunction in patients with cardiomyopathy. To identify candidate genes, we searched for gene signatures associated with arrhythmias and then examined the target genes of miR-130 and miR-95 within these signatures (Supplementary File S3). This approach led us to identify two genes of interest: CALM1, a target of miR-95, and TSC1, a target of miR-130. Both genes are involved in the physiopathology of cardiomyocytes and have been previously linked to ventricular arrhythmias (32, 33). These findings suggest that miR-130 and miR-95 target genes may play a role in the development and progression of cardiac dysfunction in patients with cardiomyopathy, particularly in relation to arrhythmias.

**Figure 6:**
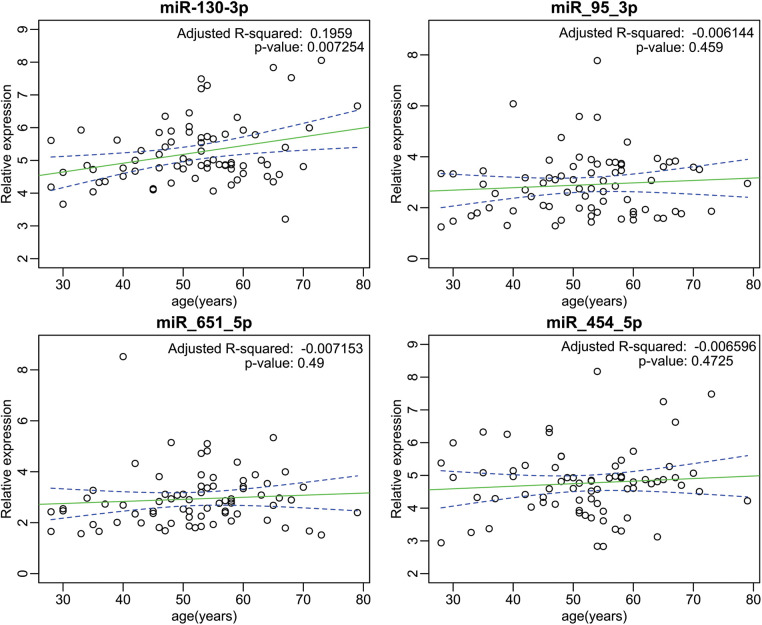
Correlation scatter plot depicting the relationship between age and upregulated miRNA expression in the G3 (CHCM). The scatter plot portrays the correlation analysis between the relative expression levels of upregulated miRNAs and age within the G3 (CHCM). The left-upper panel shows the specific case of miR-130-3p, which exhibits a discernible positive correlation with age (*p* < 0.05). While the relationship between the predictor variables and the outcome variable is statistically significant, the *R*-value suggests that only a small percentage of the variability in the outcome variable can be explained by the predictor variables included in the model.

To investigate potential associations between miRNA expression and other risk factors in G3 (CHCM) participants, we analyzed the expression levels of miRNAs in relation to gender, presence of arrhythmias, hypertension, and age (Supplementary File S4). Our analysis revealed that miR-95-3p was significantly upregulated in male participants compared to female (Supplementary Figure S1). This finding suggests a potential gender-specific effect of miR-95-3p in the context of cardiomyopathy. Furthermore, we observed a positive correlation between miR-130b-3p expression levels and age (see Figure 6, upper-left panel). While the relationship between the predictor variable and the outcome variable is statistically significant (*p* < 0.05), the *R*-value (*R* square adjusted <0.3) suggests that only a small percentage of the variability in the outcome variable can be explained by the predictor variable included in the model. Nonetheless, this outcome prompts attention towards miR-130b-3p, as it potentially links with age-related alterations in cardiac function. This finding underscores the need for a more extensive investigation to comprehend the role of miR-130b-3p in the context of age-related cardiac changes.

## Discussion

In this study, we characterized the profile of circulating miRNAs in participants with CHCM by massive sequencing techniques. We found ten differentially expressed miRNAs in subjects with CHCM. The upregulation of miR-95-3p and miR-130b-3p in CHCM compared to controls, Chagas non-cardiomyopathy, and iCM suggests that those miRNAs may be a distinguishing feature of CHCM. Examination of the gene targets linked to cardiac damage revealed genes implicated in developing arrhythmias, cardiomegaly, and hypertrophy. miR-95-3p was associated with the male gender, while miR-130b-3p showed a positive correlation with age in the CHCM group.

These findings contributed to understanding the molecular scenario in CHCM and added to well-known clinical evolution. In CHCM, 5 miRNAs linked to heart failure were identified from cardiac tissue samples. In advanced CHCM, dysregulation of the expression of miRNAs involved in gene regulation of cardiac development is observed (34). While in CHCM, some circulating miRNAs have been characterized that would be characteristic of this pathology (35), massive sequencing would allow us to know and analyze a broader profile of miRNAs involved. Although their primary location is the cell cytoplasm, they also circulate in the blood and can affect distant target tissues (17, 19, 36).

Some studies in animal models, as well as others carried out in patients with CHCM, has identified patterns of altered miRNAs expression in cardiac tissue. In mice studied during the acute phase of the disease after infection with *T. cruzi*, six miRNAs were detected accompanied by changes in clinical parameters (miR-149-5p, miR-21-5p, miR-145-5p, miR-142-5p, miR-133, miR-208) (37). Studies in CHCM subjects revealed that miR-1, miR-133a-2, miR-133b, miR-208a, and miR-208b were differentially expressed in cardiac tissue compared to healthy controls (34). Previous studies on circulating miRNAs evaluated clinical parameters and expression patterns of 6 miRNAs (miR-19a-3p, miR-21-5p, miR-29b-3p, miR-30a-5p, miR-199b-5p, and miR-208a-3p). Three of them (miR-19a-3p, miR-21-5p, and miR-29b-3p) were differentially expressed in patients with CHCM compared to Chagas non-CHCM subjects (35). Although the study showed the association of circulating miRNAs in CHCM, the analysis was limited to some pre-specified miRNAs. Recently, Gomez Ochoa et al. suggested that miR-223-5 expression levels are associated with the severity of CHCM in patients (1).

In this study, we explored the complete pool of circulating miRNAs in CHCM participants. We found 10 differentially expressed miRNAs in CHCM participants compared to healthy controls, which have not been associated with CHCM in the literature. The miR-95-3p, miR-651-5p, miR-130b-3p and miR-454-5p were upregulated; while miR-7-1-3p, miR-548au-5p, miR-3940-3p, miR- 324-5p, miR-1270 and miR-381-3p were downregulated. The miRNAs miR-95-3p and miR-130b-3p were consistently upregulated in CHCM participants compared to the other groups, suggesting that both miRNAs might be a hallmark of CHCM. We found that miR-3940-3p was also downregulated in Chagas non-CHCM participants, therefore, this miRNA could have prognostic value for progression to CHCM. This miRNA was not present in the iCM group. Other studies have shown that miR-3940-3p was differentially expressed in the serum of patients with rheumatic heart valve disease associated with augmentation of the IL1 pathway, mediating high-throughput next-generation sequencing (38). The miR-324-5p is downregulated in Chagas and ischemic cardiomyopathies, suggesting that this miRNA could be a prognostic marker of cardiomyopathy, regardless of its etiology.

This profile of microRNAs does not include those previously reported by the other CHCM studies. However, differences might be due to the techniques used to determine the expression levels of these non-coding RNAs. The most common techniques to identify miRNAs are quantitative PCR (qPCR), microarrays of miRNAs (microarrays), and bulk sequencing (NGS). In contrast to the techniques applied in other publications, mass sequencing provides a wealth of information on the profile of numerous miRNAs with the possibility of identifying new sequences (39). Another aspect that might influence the results is that detection of circulating miRNAs can vary within serum and plasma. For example, Vasques Nonaka et at reported increased expression levels of miR-19a-3p, miR-29b-3p, and miR-30a-5p in serum samples compared to plasma from the same subjects (35).

The search for target genes of differentially expressed miRNAs in CHCM yielded candidates associated with hypertrophy, atrial and ventricular arrhythmias, and dilated cardiomyopathy. Our clinical data showed a high incidence of arrhythmias in subjects with CHCM. We found that genes CALM1 and TSC1 are modulated by miR-95-3p and miR-130b-3p, respectively. Dysregulation of protein products of CALM1 and TSC1 has been associated with arrhythmia (32, 33) and might be relevant in the arrhythmogenesis processes of CHCM.

Herein, one interesting finding is the association of the level of specific miRNAs expression to gender and age. Other studies suggest that miRNA transcriptome differs between males and females, and, in disease, some deregulated miRNA might be oppositely expressed (40). Sexual dimorphism, related to clinical parameters, is well-recognized for cardiac diseases like ischemic, hypertrophic, or dilated cardiomyopathy. Similarly, miRNA profiles in heart disease show significant sex differences that might influence cardiac performance (41). In the CHCM group, miR-95-3p showed higher expression in male subjects. This finding might be relevant to explore in the future, with confirmatory studies in a broad sample.

Moreover, we found that miR130b-3p positively correlated with age in CHCM participants. This finding is in concordance with the role of miRNAs in aging. Various miRNAs are involved in the development of cellular senescence and associated pathways to aging (42). Previous studies observed that circulating miR-130a and b are implicated in age-related cardiovascular disease and processes like cardiac fibrosis and hypertension (43). In the CHCM group, miR-130b-30 might play a role in the influence of aging on CHCM. However, a limitation of the study is that no adjustments were made in the analysis of the miRNAs for confounding variables, such as sociodemographic and clinical characteristics.

This study is exploratory, and the differential expressions identified should be confirmed and validated by other techniques and in more extensive studies. Increasing sample size might provide new insights related to differential levels of up and down-regulated miRNAs in the CHCM and the possible association of some of them to other risk factors like gender and age.

## Conclusions

Our data suggest that the expression of circulating miRNAs in serum could be associated with different cardiac phenotypes in CHCM subjects, compared with Chagas non-CHCM, iCM and healthy controls. These could contribute to broadening the search for more evidence from the new miRNAs identified and open multiple pathways of exploration for their validation and translation to clinical practice.

## Data Availability

The datasets presented in this study can be found in online repositories. The names of the repository/repositories and accession number(s) can be found in the article/**Supplementary Material**.
