# Supplementary material for: Circulating microRNAs as biomarkers of Chagas cardiomyopathy
Source: Front Cardiovasc Med. 2023 Dec 19;10:1250029. doi: 10.3389/fcvm.2023.1250029 (PMC10762800; doi:10.3389/fcvm.2023.1250029)
Supplement: Supplementary file 5 [file Datasheet1.docx]

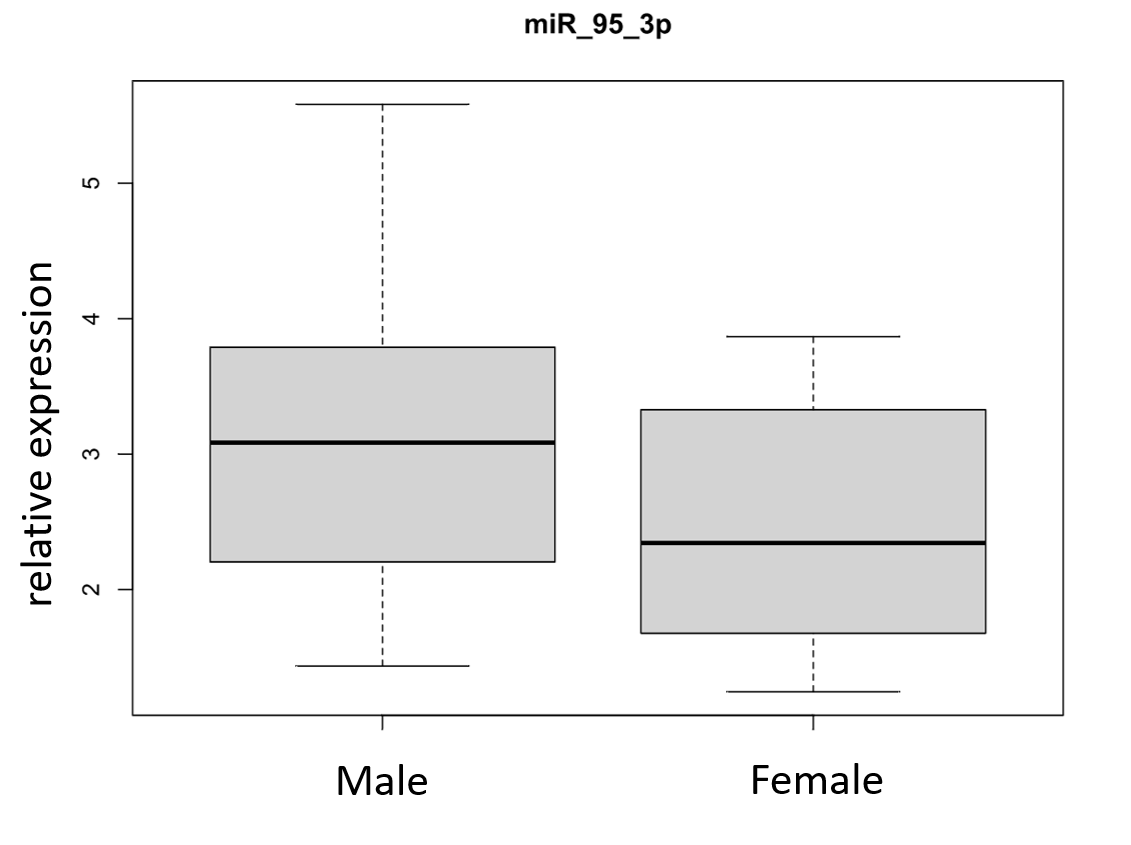
Supplementary Figure 1: Relative expression of miR-95-3p is augmented in males compared to females subjects.

Values of relative expression miR-95-30 were compared between male and female subjects of the G3 group (CHCM). Differences were significant according to T-test p < 0.05.
